# Seasonal variation of carbon fluxes in a sparse savanna in semi arid Sudan

**DOI:** 10.1186/1750-0680-3-7

**Published:** 2008-12-01

**Authors:** Jonas Ardö, Meelis Mölder, Bashir Awad El-Tahir, Hatim Abdalla Mohammed Elkhidir

**Affiliations:** 1 Physical Geography and Ecosystems Analysis, Lund University, Sölvegatan 12, S-223 62 Lund, Sweden; 2 Agricultural Research Cooperation, El Obeid Research Station, P.O. Box 429, 51111, El Obeid, Sudan

## Abstract

**Background:**

Large spatial, seasonal and annual variability of major drivers of the carbon cycle (precipitation, temperature, fire regime and nutrient availability) are common in the Sahel region. This causes large variability in net ecosystem exchange and in vegetation productivity, the subsistence basis for a major part of the rural population in Sahel. This study compares the 2005 dry and wet season fluxes of CO_2 _for a grass land/sparse savanna site in semi arid Sudan and relates these fluxes to water availability and incoming photosynthetic photon flux density (PPFD). Data from this site could complement the current sparse observation network in Africa, a continent where climatic change could significantly impact the future and which constitute a weak link in our understanding of the global carbon cycle.

**Results:**

The dry season (represented by Julian day 35–46, February 2005) was characterized by low soil moisture availability, low evapotranspiration and a high vapor pressure deficit. The mean daily NEE (net ecosystem exchange, Eq. 1) was -14.7 mmol d^-1 ^for the 12 day period (negative numbers denote sinks, i.e. flux from the atmosphere to the biosphere). The water use efficiency (WUE) was 1.6 mmol CO_2 _mol H_2_O^-1 ^and the light use efficiency (LUE) was 0.95 mmol CO_2 _mol PPFD^-1^. Photosynthesis is a weak, but linear function of PPFD. The wet season (represented by Julian day 266–273, September 2005) was, compared to the dry season, characterized by slightly higher soil moisture availability, higher evapotranspiration and a slightly lower vapor pressure deficit. The mean daily NEE was -152 mmol d^-1 ^for the 8 day period. The WUE was lower, 0.97 mmol CO_2 _mol H_2_O^-1 ^and the LUE was higher, 7.2 *μ*mol CO_2 _mmol PPFD^-1 ^during the wet season compared to the dry season. During the wet season photosynthesis increases with PPFD to about 1600 *μ*mol m^-2^s^-1 ^and then levels off.

**Conclusion:**

Based on data collected during two short periods, the studied ecosystem was a sink of carbon both during the dry and wet season 2005. The small sink during the dry season is surprising and similar dry season sinks have not to our knowledge been reported from other similar savanna ecosystems and could have potential management implications for agroforestry. A strong response of NEE versus small changes in plant available soil water content was found. Collection and analysis of flux data for several consecutive years including variations in precipitation, available soil moisture and labile soil carbon are needed for understanding the year to year variation of the carbon budget of this grass land/sparse savanna site in semi arid Sudan.

## Background

Much carbon cycle research has focused on temperate and tropical forests whereas savannas have been less frequently studied [1]. Savanna ecosystems cover a larger area than any forest biome [2] and are a potential carbon sink [3-6] due to losses of vegetation and soil organic carbon during the last century. Taylor and Lloyd [7] estimate that 15% of the annual global carbon sink might be attributable to savannas and seasonally dry tropical forest ecosystems. Grace et al [6] report an average carbon sequestration rate of 14 g C *m*^-2 ^*year*^-1 ^for tropical savannas, a rate that may increase in areas protected from fire, grazing and intense cultivation [8]. Increased water use efficiency (WUE) due to higher atmospheric CO_2 _content may further enhance this potential [9]. Carbon sequestration in biomass and soils have been proposed as an attractive strategy for addressing the UN convention on desertification (UNCCD) in degraded semi-arid ecosystems [3,5,10] while simultaneously contributing to the UN Framework Convention on Climate Change (UNFCCC) in the context of improving soil resources [11]. These benefits include increased soil fertility [12,13], counteracting land degradation [14], reducing atmospheric CO_2 _concentration [15], as well as secondary social and economic effects despite obstacles [16,17]. These obstacles include unequal possibilities to participate in sequestration programmes due to the economic situation of poor landholders that also often have the most degraded soils (Ardö, unpublished). Additional obstacles include low sequestration rates in semiarid areas, resulting in relatively high monitoring costs and institutional difficulties, all limiting participation in, and success of, carbon sequestration and carbon trading programs [18].

**Figure 1:**
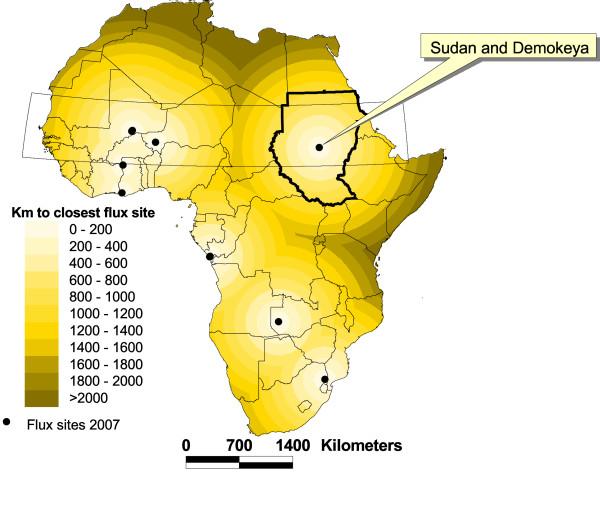
Study site, Sahel and proximity to other flux sites in Africa. Eleven sites (all not visible as some are located very close to each other) that recorded fluxes of CO_2 _in 2007 are shown and the distance to the closest site in km. The rectangle indicate the Sahel region, 10 – 20°N.

In 2007 there were 11 active sites measuring CO_2 _fluxes in Africa. This includes sites from Fluxnet [19], 20 June 2007], the CARBOAFRICA project [20], the AMMA project [21] and one site in Burkina Faso [22]. Five of these eleven sites are located in the Sahel region (defined as 10–20 degrees N, see Figure 1). Older flux measurements in the Sahel include in the HAPEX experiment [23-25] in Niger and measurements in Burkina Faso in 1996–1997 [26]. Of these 11 African sites, three are in savanna and two in grassland areas. The remaining sites are located in cropland (two sites), evergreen forest (one site), Eucalyptus plantation (one site) and woodland (two sites).

Currently (2007) CO_2 _flux measurements in the Sahel include one site in the Sudan (Demokeya, Sudan, the site presented here), one site in Burkina Faso [22], and some sites within the AMMA project [21]. Several other sites have, or are soon to have eddy covariance measurements, or meteorological and heat flux measurements within the EU-funded CARBOAFRICA-project [20]. Figure 1 illustrates flux sites with confirmed data collection in 2007.

The number of available CO_2 _flux measurements in Africa is low compared to the number of sites estabished in Asia, Europe and the Americas. Africa, with an area of 30 million km^2 ^is covered by approximately 11 sites, whereas North America is 24 million km^2 ^and covered by ≈172 (132 in USA, 38 in Canada and two in Mexico) measuring CO_2 _flux. Even if the number of sites is not constant over time, the density of flux towers is approximately 35 times higher in North America compared to Africa.

In a similar manner are the flux sites located in savannas under-represented in relation to the area covered (especially compared to sites in forests) and the need for more flux towers in savannas as well as in areas with low spatial coverage have been identified [22,27,28]. The low flux measurement station density in Africa is accompanied by a low density network of climate stations, averaging one station per 26000 km^2^, eight times lower than the WMO's (World Meteorological Organisation) recommendation [29]. The number of climate monitoring stations in Africa has decreased since the 1970's [30]. Understanding climate, climate change as well as present and future effects and risks, aspects of mitigation and adaptation and carbon cycle studies in Africa are important issues. These issues would benefit from denser measurements, both of fluxes as well of standard meteorological data [31,32].

As an attempt to contribute to filling in these data gaps, we established meteorological and flux measurements in central Sudan (Figure 1), in the Sahel, a region where recent vegetation increases have been observed [33-35]. Precipitation has been identified as the primary driver of these vegetation changes [36], yielding a net gain of carbon in Sahel during 1980's and 1990's [37]. Factors such as migration, armed conflicts [34], pasture and cropping intensity may also contribute to observed vegetation changes in the Sahel, even if the human footprint found was weak [38]. Satellite based studies of vegetation phenology report significant positive trends for the length of the growing season and for the timing of the end of the growing season for the Soudan and Guinean regions [39]. Verification of the satellites observed changes is difficult on the local scale [40,41], partly due to differences in spatial resolution of the satellite data used (NOAA AVHRR, MODIS) and field observations.

Estimation of gross primary production using remotely sensed data and the light use efficiency (LUE) methodology is a widely applied concept for spatially continuous modeling of the carbon cycle [42-44]. This concept can be a powerful tool for estimating net ecosystem CO_2 _exchange with high temporal and spatial resolution [45]. Locale scale validation and operationalization of these models can be performed using eddy covariance data [46], especially at sites where incoming and reflected photosynthetic photon flux density PPFD or absorbed PPFD is measured. WUE, the amount of carbon fixed per amount of water used, has been used as an indicator of desertification [47,48] is assumed to increase with increasing atmospheric CO_2 _concentration [9].

**Figure 2:**
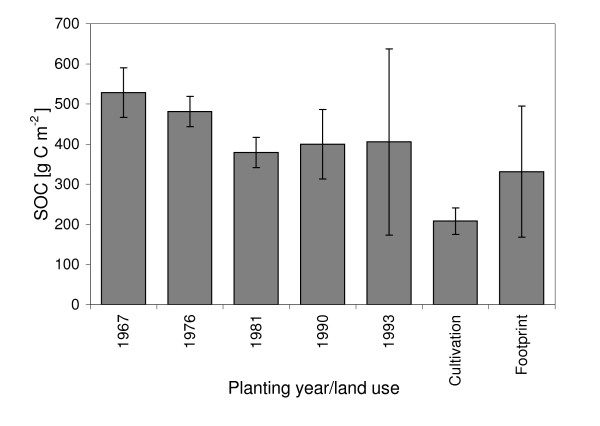
Soil organic carbon in the upper 20 cm of the soil profile in *Acacia senegal *plantations with different stand age, at a cultivated site and in the close vicinity of the flux tower (foot print). Error bars indicate ± 1 standard deviation, four samples at each site, all samples from Demokeya.

Some of the first data obtained from measurements at the Sudanese site is presented below as a short, mainly descriptive and comparative study of CO_2 _fluxes during the dry and wet season of 2005 (see additional file 1 and 2). This project is an continuation of earlier work on soil carbon sequestration [5,8,49], (Figure 2).

This study aims to provide information on carbon dynamics in a semi arid savanna through measurements of fluxes of CO_2_, water vapour and energy in central Sudan. Specifically we aim to: 1) quantify CO_2 _flux during wet/dry season, and 2) relate CO_2 _fluxes to water availability and incoming photosynthetic photon flux density (PPFD).

## Results

**Figure 3:**
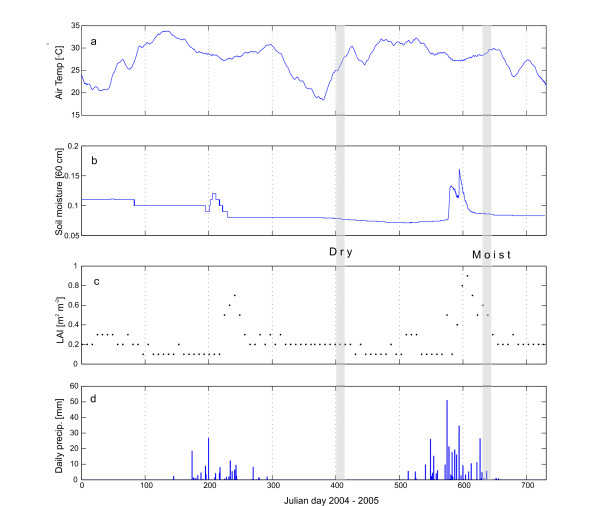
Air temperature (30 day running mean) (a), volumetric soil moisture (b), LAI from MODIS (c) and daily sum of precipitation (d) from January 1, 2004 to December 31, 2005. Shaded areas indicate times of CO_2 _flux measurements during the dry and wet (Moist) season.

**Figure 4:**
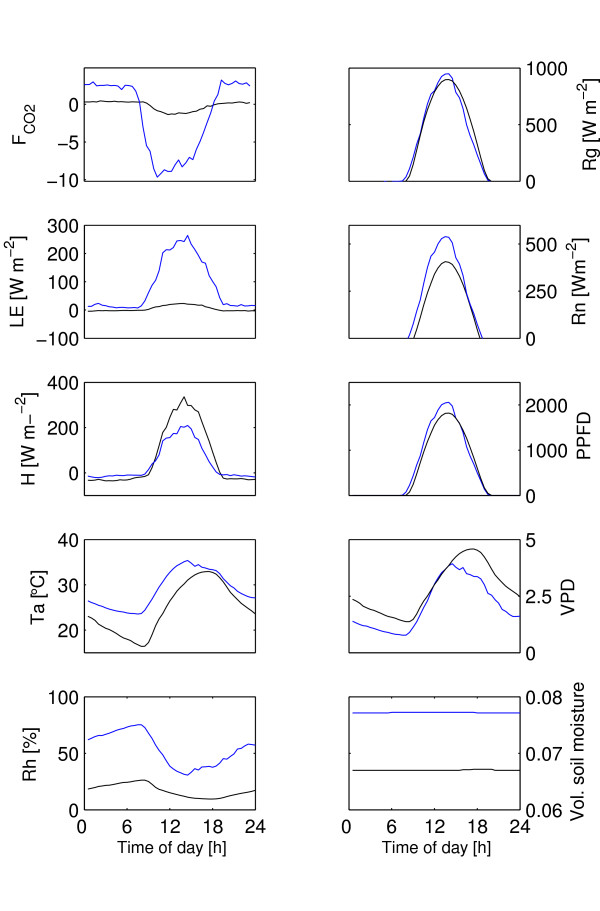
Mean diurnal fluxes and meteorological variables representative for the dry and wet season 2005 in Demokeya, the Sudan. Blue lines for the wet season and black lines for the dry season. $F_{CO_{2}}$ = CO_2 _flux, [*μ*mol CO_2 _m^-2 ^s^-1]^, LE = Latent heat, H = Sensible heat, Ta = Air temperature, Rh = relative humidity, Rg = Global radiation, Rn = Net Radiation, PPFD = photosynthetic photon flux density [*μ*mol m^-2 ^s^-1^], VPD = Vapour pressure deficit [KPa] and Volumetric soil moisture content [fraction]. Based on averages of 30 min. samples for each period.

The temporal variation of the general plant growth driving forces (temperature, precipitation, soil moisture) and LAI during 2004 and 2005 show the distinction between the dry and wet seasons (Figure 3). The average "dry" and "wet" day clearly differs in most aspects ($F_{CO_{2}}$, LE, H, vapor pressure deficit (VPD), relative humidity (RH), global radiation (Rg), PPFD, air temperature (Ta) and soil moisture), (Figure 4).

### Dry season

**Figure 5:**
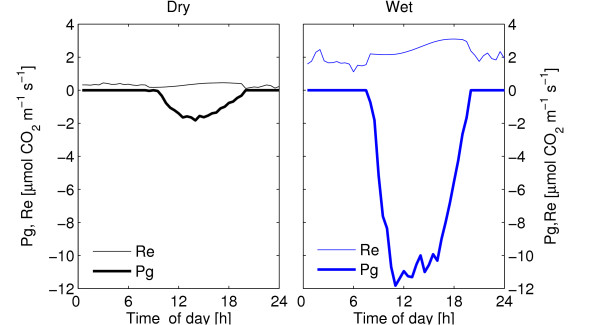
Mean diurnal ecosystem respiration (Re) and and gross primary production (Pg) for the dry and wet season 2005. Based on averages of 30 min. samples for each period.

**Figure 6:**
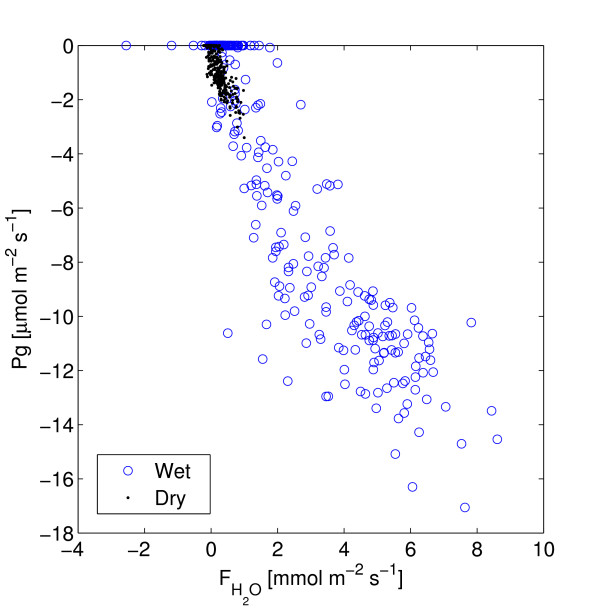
Pg versus $F_{H_{2}O}$. Based on averages of 30 min. samples for each period.

**Figure 7:**
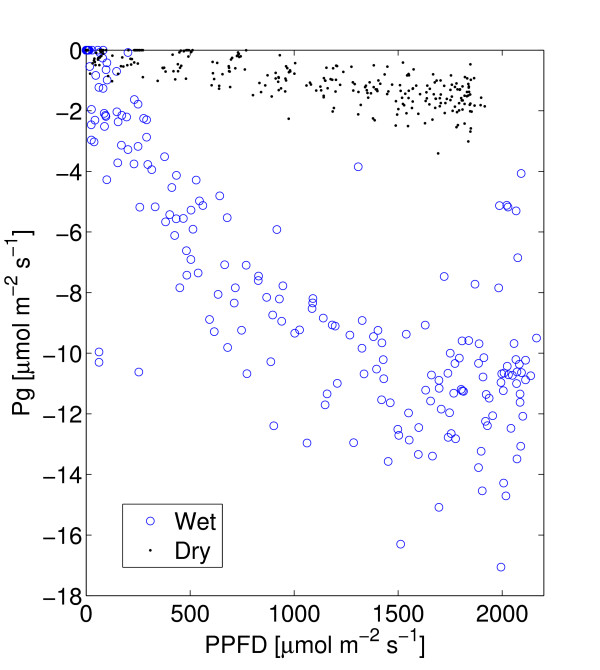
Light use response curves for the dry and wet season. Based on averages of 30 min. samples for each period.

The dry season is characterized by low soil moisture availability (6.7%, which gives an plant available water content of approximately 1.7% assuming a wilting point of 5%), low evapotranspiration and a high vapor pressure deficit (Figure 4). The mean daily NEE was -14.7 mmol CO_2 _d^-1 ^during the dry season (Table 1, Figure 5). Mean daily gross primary production (Pg) was -42.3 mmol CO_2 _d^-1 ^and ecosystem respiration (Re) was 27.6 mmol CO_2 _d^-1^. WUE was 1.6 mmol CO_2 _mol H_2_O^-1 ^and LUE was 0.95 mmol CO_2 _mol PPFD^-1 ^(Table 1, Figures 6, 7). Pg was a weak, but linear function of PPFD (*R*^2 ^= 0.78, Figure 7). Peak PPFD reached 1800 *μ*mol m^-2 ^s^-1 ^during mid-day (Figure 4).

**Table 1:** Summary results for the dry and wet season 2005, sd = standard deviation.

Variable	Unit	Dry (sd)	Wet (sd)
n days	-	12	8

Mean daily *LE*	Wm^-2^	4.8 (2.9)	82.5 (11.0)
Mean daily *H*	Wm^-2^	64.6 (7.3)	41.9 (7.3)

Mean daily *P*_*g*_	mmol CO_2 _m^-2 ^d^-1^	-42.30 (7.9)	-344.40 (56.85)
Mean daily *R*_*e*_	mmol CO_2 _m^-2 ^d^-1^	27.60 (2.3)	191.30 (12.01)
Mean daily NEE	mmol CO_2 _m^-2 ^d^-1^	-14.70 (9.6)	-152.40 (53.54)

Water use efficiency, ($F_{CO_{2}}$/$F_{H_{2}O}$)	mmol CO_2 _mol H_2_O^-1^	1.597	0.967
Mean daily LUE (ϵ)	mmol CO_2 _mol PPFD^-1^	0.954	7.196
*R*^2 ^Pg vs PPFD (linear function)	-	0.78	0.82

### Wet season

The wet season was, compared to the dry season, characterized by slightly higher soil moisture availability, (7.8%), which led to a plant available water content of approximately 2.8%, higher evapotranspiration and a slightly lower vapor pressure deficit (Figure 4). The mean daily NEE (152 mmol CO_2 _m^-2 ^d^-1^) was approximately ten times higher compared to the dry season (Table 1, Figure 4). Mean daily Pg was -344.4 mmol CO_2 _d^-1 ^and Re was 191.3 mmol CO_2 _d^-1^. WUE was lower, 0.97 mmol CO_2 _mol H_2_O^-1 ^and the LUE was higher, 7.2 *μ*mol CO_2 _mmol PPFD^-1 ^during the wet season compared to the dry season (Table 1, Figures 6, 7). During the wet season, Pg increased with PPFD to about 1600 *μ*mol m^-2^s^-1 ^(Pg as a quadratic function of PPFD yields *R*^2 ^= 0.90, Pg as a linear function of PPFD yields *R*^2 ^= 0.82) and then levels off (Figure 7). Peak PPFD reached >2000 *μ*mol m^-2 ^s^-1 ^during mid-day (Figure 4).

### Dry to Wet transition

**Figure 8:**
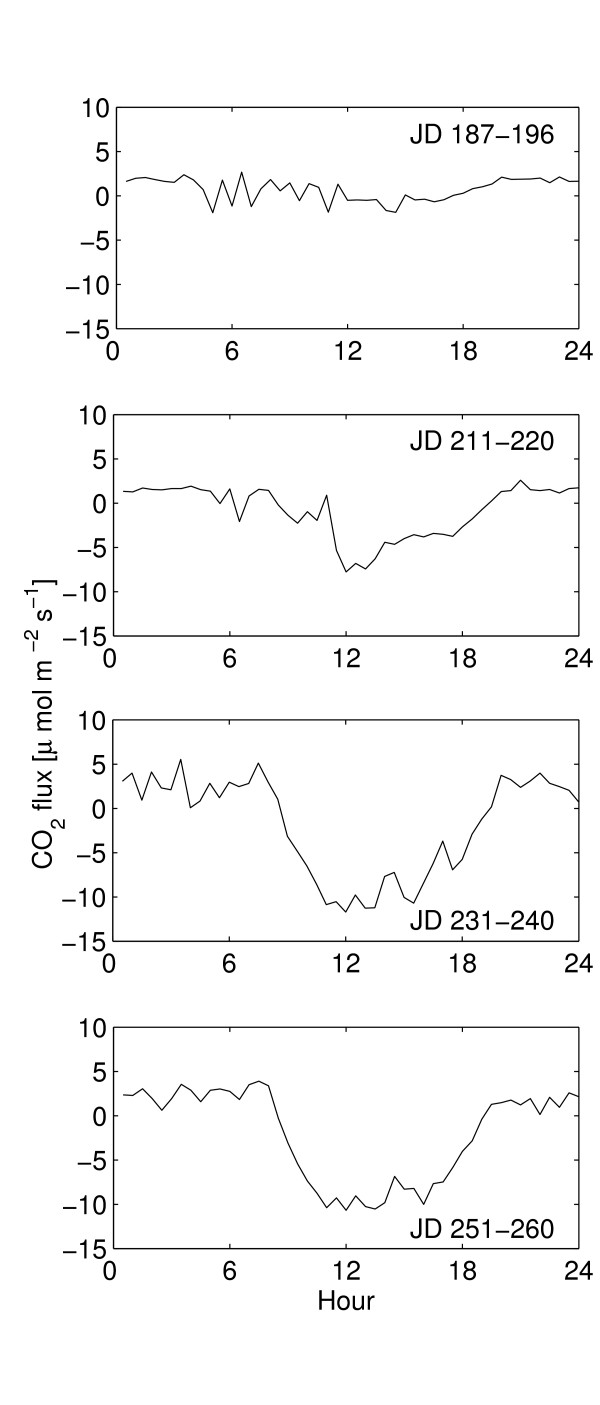
Transition from dry to wet season 2007. Each graph display the mean diurnal CO_2 _flux for a 10-day period. Based on averages of 30 min. samples for each period.

As a complement to the dry and wet season data presented above, flux data ($F_{CO_{2}}$) from JD 187 to JD 265 for 2007 were used (Soegaard et. al, unpublished) to illustrate the transition from the dry to wet season. These data were processed the same way as the 2005 data and originate from the same instruments. Mean $F_{CO_{2}}$ for four 10-day periods, starting on JD 187, 211, 231 and 251 were 61.5, -77.0, -162.6 and -179.2 mmol CO_2 _m^-2 ^d^-1 ^respectively (Figure 8). The first period (JD 187 – JD 196 2007, data not shown), occurring just after the first major precipitation events, shows a source of CO_2_. During the second period, a sink starts to develop as the assimilation by the field layer (grasses and herbs) increases. During the third and fourth period the sink becomes stronger as grass biomass and cover increases. Volumetric soil moisture was around 10% during this period (JD 187-JD 265, 2007) resulting in slightly more (ca. 5%) plant available water compared to the wet season 2005. The annual precipitation in 2007 was ca 365 mm. i.e. a normal year, compared to the 350 mm that fell during 2005 and compared to the long term average of approximately 320 mm y^-1^.

### Energy balance

**Figure 9:**
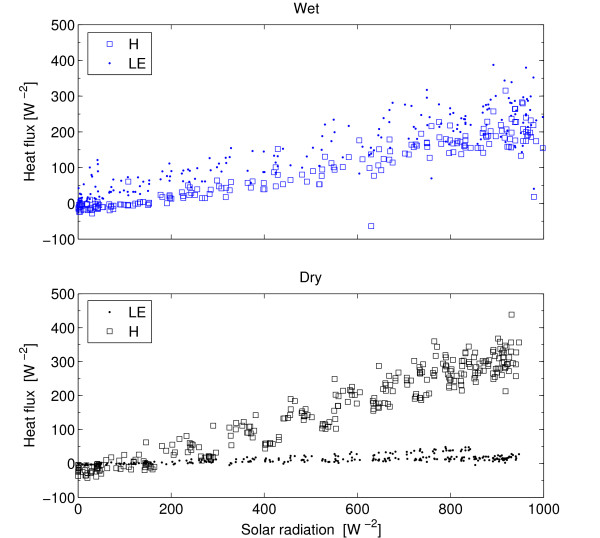
Energy partitioning of sensible heat (H) and latent heat (LE) versus incoming solar radiation during the dry (below) and wet season (above). Based on averages of 30 min. samples for each period.

**Figure 10:**
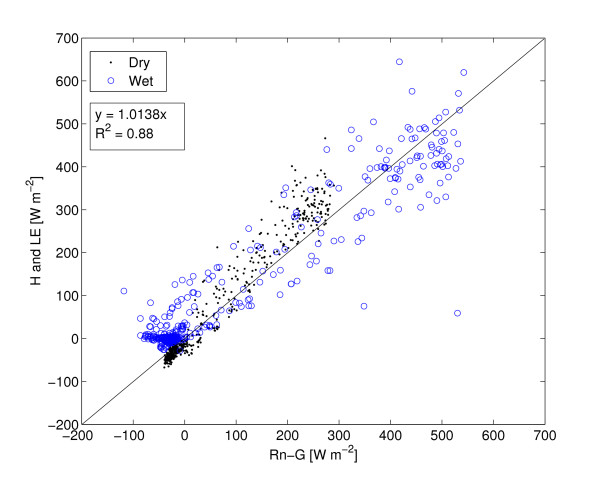
Energy balance closure for the dry and wet season. Rn = net radiation, G = soil heat flux, H = sensible heat and LE = latent heat. The line shows a 1:1 ratio. Based on averages of 30 min. samples for each period. Regression offset forced to 0.

Maximum incoming solar radiation was 1000 *Wm*^-2 ^during the wet season and 950 *Wm*^-2 ^during the dry season (Figure 4). Due to differences in available moisture LE was low during the dry season (max 50 *Wm*^-2^) compared to the wet season (max 390 *Wm*^-2^). Both LE and H are linear functions of Rg during the wet season whereas only H shows a linear dependence on Rg during the dry season (Figure 9). Canopy heat storage was not included as it is of minor importance due to low height and density of the trees [50]. Comparing H+LE with Rn-G reveals a strong linear relation with an offset of 16.8, (*H *+ *LE *= 0.97 * *Rn - G *+ 16.8 *r*^2 ^= 0.89, *n *= 960, in Figure 10 is the regression offset forced to 0 and hence slightly different), comparable to results reported by Wilson *et al*. [50]. The energy balance closure method as measured by the eddy covariance system confirms that the measurements are of good quality (Figure 10).

## Discussion

### Fluxes

As expected, dry season fluxes were small with substantially lower Pg, Re and NEE compared to the wet season (Table 1, Figure 5). Wet season NEE was about ten times larger than during the dry season NEE. More surprisingly, a small assimilation (-0.2 g C m^-2 ^day^-1^) and corresponding latent heat flux was observed during the dry season (Table 2, Figure 4).

**Table 2:** Net ecosystem exchange in similar environments.

Site	Vegetation	MAP	NEE [*g C m*^-2^]					Ref.
			Annual	Monthly		Daily		
				
				Dry	Wet	Dry	Wet	
					
Baja	Desert shrub	174	-39, -52	0.7 – 25	-12 to -41	0.4^1^	-0.48^2^	[28]
Demokeya	Sparse savanna	320	-	-	-	-0.2	-1.8	This study
Maun	Savanna woodland	464	12	-	-	1.2 – 2.4	-0.6 to -2.4	[1]
Hapex	Sahelian fallow savanna	495	-32	-	-	≈0.3	0.6 to -3.6	[23]
Virgina Park	Open woodland savanna	667	44^1^	≈6	14 to -52			[57]
Bontioli	Shrub dominated savanna	926	-179, -429	5–20^3^	-35 to -175^3^	0.2–0.4	-1.4 to -5.9	[22]
Aguas Emendadas	Cerrado	1500	-	-	-	0.6	-1.2	[60]

MAP, Mean Annual Precipitation, mm yr^-1^.

NEE – negative values denote uptake.

^1 ^Not presented in paper calculated as Monthly NEE/30.

^2 ^Averaged for the period July 2001–March 2003.

^3 ^Estimated from Fig. 4a in [22].

During the dry season, the volumetric water content of the upper 60 cm of the soil was 6.7% (Figure 4), just above the minimum soil moisture content recorded during 2005, which was 6.2%. Assuming a wilting point of 5–7%, (depending on calculation method [51,52]), resulted in a estimated plant available water content (PAWC) of ≈0–1.7% during the dry season and PAWC of ≈1.0–2.7% during the wet season. This minor difference in plant available water seem to be crucial for plant growth and also determines the seasonality for herbs and grasses, which in savanna ecosystems are adapted to short periods with sufficient water for growth and reproduction [53].

The dry season assimilation when the field cover is dry but Acacias keep some green leaves, and the upper two m of the soil profile has a low PAWC could potentially be explained by the effect of Acacias with access to deep water reserves [54-56]. The deep root system of *Acacia senegal *allows efficient use of soil water resources and the presence of this tree may thus enhance the productivity of the whole system compared to a system without trees [56]. Leuning *et al*. report maintained annual transpiration above rainfall during dry conditions in Eucalypt forests of Australia and explain it by access to deep water [57]. As an alternative explanation, Acacias could potentially store water in trunks or in xeromorphic adaptations to dry environments [58]. Hence more than one process may be involved in allowing assimilation and transpiration during dry periods. Soegaard *et al *[26] stressed the importance of soil moisture in a study in a similar environment in Burkina Faso. They reported that midday radiation load and leaf temperature are the most important parameters for predicting carbon assimilation and they state '*At the end of the growing season when soil moisture contents is low (< 8 vol. %) the rate of carbon assimilation is also limited by the stomatal control*'. With a volumetric soil moisture content of 7.8% (Figure 4), for the wet season data, we can assume the corresponding flux to be partly limited by soil moisture, with peak assimilation occurring earlier in the season when soil moisture was higher (no flux data available, cf. Figure 3).

Ecosystem respiration (Re) was low during the dry season, 0.2–0.3 *μ*mol CO_2 _m^-2 ^s^-1 ^due to low soil moisture, and due to low LAI [59] as the field layer was dry. Wet season Re was ca. 2 *μ*mol CO_2 _m^-2 ^s^-1^, when soil moisture and LAI was slightly higher (Figures 2, 4 and 5).

Observed fluxes of CO_2 _and H_2_O are similar to fluxes reported in studies of similar environments (Table 2), except for the small assimilation observed during the dry season. Seasonal and interannual variability, as well as the short periods of data available for some sites, decrease the opportunity for meaningful intercomparison among different sites. Hastings et al [28] reported annual NEE of -39 and -52 [g C m^-2^], in two different years, for a dry desert shrub community in California, proposing water storage in stems and roots to act as buffers to variations in annual rainfall. Dividing the monthly NEE presented by Hastings et al [28] with 30 gives a mean daily sink of 0.48 g C for the wet season and a mean daily source of 0.4 g C for the dry season. This indicates lower NEE during wet conditions compared to this study which is reasonable due to the lower moisture availability. Veenendaal et. al [1] studied fluxes of CO_2 _from a broad-leaved (Mopane) semi-arid savanna in southern Africa and reported a net uptake of 0.6–2.4 g C m^-2 ^d^-1 ^during the latter part of the wet season (March, 1999). During the dry season, the sink progressively turned into a source of 1.2–2.4 g C m^-2 ^d^-1^. This site was however wetter (mean annual precipitation (MAP) = 464 mm) and had a higher tree density and canopy cover (35–40%) compared to the site presented here. The net annual accumulation reported was 12 g C and the daily wet season NEE is of the same range as in this study (Table 2). Hanan et. al [23] combined rainy season measurements with dry season simulations for a Sahelian fallow savanna and reported an annual net ecosystem uptake of 32 g C m^-2^. Growing season (JD 150–285) net ecosystem uptake was 96 g C m^-2^, resulting in a mean daily uptake of around 0.7 g C m^-2 ^d^-1 ^for the growing season and a peak uptake around 4 g C m^-2 ^d^-1^. During the dry part of the year the system was a small carbon source (approximately 0.3 g C m^-2 ^d^-1^).

Leuning [57] reported an annual NEE of 44 g C m^-2 ^for a open woodland in Australia (MAP = 667 mm). Mean monthly NEE was around 6 g C m^-2 ^month^-1 ^during the dry season and varied from a source of 14 to a sink of -52 g C m^-2 ^month^-1 ^during the wet seasons, with a large interannual variability. Brümmer et. al studied a shrub dominated savanna in Burkina Faso and reported annual uptake of 179 and 429 g C m^-2 ^yr^-1 ^for two consecutive years. Mean monthly fluxes were 5–20 g C m^-2^month^-1 ^during the dry season and -35 to -175 g C m^-2 ^month^-1 ^during the wet season. Large interannual variation was observed for the wet season while the dry season showed only minor variations. In a Brazilian cerrado savanna, mean daily NEE of around 0.6 and -1.2 g C m^-2 ^d^-1 ^were observed for the dry and wet periods respectively [60]. Common for all these sites (Table 2) are low dry season fluxes, with NEE mostly around 0.2–0.6 g C m^-2 ^d^-1^, except for one site with higher NEE (Maun, [1]). Wet season NEE range from -0.5 to -5.9 g C m^-2 ^d^-1^. Interannual and intraseasonal variation is large and varying definitions on when dry/wet seasons start and end affect comparability. Observed wet season fluxes are well within the ranges of similar sites (Table 2).

### Transition

During the transition from the dry to the wet season (2007), the system turns from a being a source of 58 mmol CO_2 _m^-2 ^d^-1 ^in early July to a sink of 166 mmol CO_2 _m^-2 ^d^-1 ^in mid September (Figure 8). It takes approximately 30 days after the first significant precipitation for the system to turn from a source to a sink of CO_2_. This agrees with the findings from Southern Africa where "a strong release of CO_2 _during the early wet season" was reported [1], (cf Figure 8) and with findings from Sahel, where a net ecosystem loss occurred for 50 days from leaf emergence [23]. As the vegetation develops, both assimilation and respiration increases (precipitation was slightly higher in 2007 than in 2005). Due to the strong dependence of both photosynthesis and respiration on available soil moisture a large variability in seasonal transition and in seasonal magnitude of net ecosystem exchange of CO_2 _in the Sahel might be expected.

### Light use efficiency

*Physiological *light-use efficiency [61], which is defined as *P*_*g*_/*Q*_*abs *_where *Q*_*abs *_is the absorbed photosynthetically active radiation, is frequently used [25,62], reducing comparability to the *ecological *light-use efficiency applied here (based on PPFD and defined below). Gilmanov reported weekly maximum LUE values from 7 to 12 [mmol CO_2 _mol PPFD^-1^] for semi arid grasslands in Europe [61]. The lower range is comparable to the wet season daily averages of 7.2 [mmol CO_2 _mol PPFD^-1^] (Table 1) in this study. Moncrieff et. al [63] report light use efficiencies (30 min values) ranging from 0 to 15 [mmol CO_2 _mol PPFD^-1^] with the lower values for dryer conditions and the higher for more wet conditions, i.e. similar to the dry and wet season LUE presented in Figure 7 and similar to results from Southern Africa [1] and West Africa [22].

### Water use efficiency

The WUE (mmol CO_2 _mol H_2_O^-1^, Table 1) was higher during the dry season compared to the wet season. During the dry season we assumed that a larger fraction of the evapotranspirational flux ($F_{H_{2}O}$) was transpiration compared to the wet season when soil evaporation and canopy evaporation are more likely to occur. The overall small dry season fluxes would be more prone to measurement errors compared to the stronger signal during the wet season. Comparison among studies is partly prevented due to that WUE is sometimes defined as the ratio between biomass production and evapotranspiration and sometimes as ratio between biomass production and transpiration. Friborg et al. [25] reported an average WUE of 8.5 (range 6–14) [mg CO_2 _g H_2_O^-1^] corresponding to 3.7 (range 2.4–5.7) mmol CO_2 _mol H_2_O^-1^, but based on transpiration only and not on evapotranspiration as used here. WUE based on evapotranspiration would be lower and Moncreieff et al [63] present WUE's based on the evapotranspirational flux of water of 2–8 [mg CO_2 _g H_2_O^-1] ^for millet, 0.2–5 for fallow bush and an average WUE [mg CO_2 _g H_2_O^-1] ^of 2 for a tiger bush. This corresponds to to 0.8–3.2, 0.08–2 and 0.2 mmol CO_2 _mol H_2_O^-1 ^respectively, but show larger variation as it is 30-min averages based on daytime data only, versus the daily averages reported here (Table 1).

### Implications for management

Soil moisture strongly determines both carbon assimilation and ecosystem respiration in semi arid areas. Soil moisture amount and availability can be influenced by management (eg. by intercropping of crops and trees together in agroforestry systems). Agroforestry increases deep infiltration of water, improves soil structure [64] and increases WUE [65]. Furthermore the presence of trees allows the use of deep moisture [57] not accessible for grasses and herbs. Agroforestry systems also capture wind transported fine material and hence increases the clay and silt fractions of the soil and increase organic matter (Figure 2), water holding capacity and soil nutrient status [66]. This results in increased water holding capacity and less soil evaporation. The use of *Acacia senegal *in bush fallow or agroforestry also increases grain yield [66,67]. Agroforestry positively influences the micro climate, provides shade and produces fuel wood, the major energy source in Sahel and non-timber forest products that significantly contribute to income in rural areas [68]. Management could therefore influence the total amount of plant available water, a crucial resource in semi arid areas. Agroforestry systems on sandy soils and including Acacias with deep roots, could potentially increase the total moisture available for plant production compared to for example monocropping of Millet or deforested savannas used for grazing purposes. This is partly supported by the dry season net assmilitation obeserved (Figure 5).

## Conclusion

Fluxes of CO_2 _measured with the eddy covariance methodology during two short periods at a semi arid site in central Sudan indicate that this ecosystem was a sink for carbon both during the dry and wet season 2005. Small differences in plant available soil water content had a strong influence on CO_2 _flux. Fluxes presented here are comparable to results from similar studies in West and Southern Africa, except the small dry season assimilation attributed to Acacias with access to deep soil moisture. Due to the small assimilation observed and the small data set available it is unclear how valid this result is and analysis of additional dry season data from this site should be conducted (and is in progress). Collection of flux data for several consecutive years including variability in precipitation, available soil moisture and available soil carbon are needed for understanding the year to year variation of the carbon budget of this sparse savanna site in semi arid Sudan. These measurements can also provide useful data for validation and calibration of ecosystem models and for remote sensing studies.

## Methods

### Site description

The site (called Demokeya) is located in Kordofan, central Sudan, approximately 35 km north east of El Obeid, close to the village of Demokeya, (13.3°N, 30.5°E) (Figure 1). It is characterized by a sparse Acacia savanna (dominating species include *Acacia nilotica, A. tortilis, A. senegal*) with a canopy cover of 5–10% and a ground cover composed mainly of grasses (dominating species are the perennial *Aristida pallida*, the annuals *Eragrostis tremula *and *Cenchrus biflorus*) and some herbs. Maximum tree height is six m and the major part of the tree canopy is located between three and five m above the ground. Grasses and herbs reach a maximum height of one m. Approximately 70% of the vegetation is assumed to be C4 plants and 30% is assumed to be C3 plants. The deep sandy soil (96.5% sand and 3.5% silt) has a estimated minimum (wilting point) and maximum (field capacity) volumetric water holding capacity of 5% and 15% respectively and hence a maximum plant available water content of around 10%. Soil organic carbon at the site varies with land cover and stand age of the *Acacia senegal *plantations (Figure 2). The soil within the footprint area, has a pH of 6.7 and contains 0.11% SOC and 0.03% N in 2007. The landscape is flat but gently undulating due to stabilized parallel sand dunes with a N-S orientation. Mean annual precipitation is 320 mm with most falling from June-October. November to May is dry. Mean annual temperature is 28° C. Grazing, cultivation and forest in the close vicinity of the measurements are restricted, but the site is not fenced. The last rain event (2 mm) prior to dry season 2005 measurements (February 2005) occurred on October 18, 2004 (Figure 3). The total precipitation in 2004 was 144 mm, i.e. less then half of the long term mean precipitation. The soil was therefore very dry during the dry season with volumetric soil moisture of ~5% in the upper 2 m.

The dry season measurements originate from the middle of the dry season, more then 100 days after the last rain (Figure 3), and characterized by low soil moisture, wilted field cover and only minor green leaves on the Acacias.

Approximately 350 mm of precipitation, distributed among a normal number of rain events, fell during the summer 2005 prior to the wet season measurements presented here (September 2005). Slightly higher volumetric soil moisture (5–9%) was recorded in the upper 2 m during the wet period. Maximum volumetric soil moisture in 2005 was 16% (Julian day 227) at 60 cm depth. Figure 3 illustrates the seasonal changes in temperature, water availability and leaf area index (LAI) during 2004 and 2005. LAI data came from a standard MODIS product (MODIS 8 day composites, MOD15A2, collection 4, 1 km resolution, ), further described in [69]. LAI was 0.2 during the dry season measurements and 0.6 during the wet season measurements with a peak of 0.9 at JD 240, 2005 (Figure 3).

### Instrumentation and measurements

Measurements were conducted at two closely adjacent locations. Fluxes of CO_2_, water vapour and sensible heat were measured with an open path system (In Situ Flux Systems AB, Ockelbo, Sweden) including an open path infrared CO_2_/H_2_O analyzer (LI7500, Li-Cor, Lincoln, Nebraska) and a Gill R3 Ultrasonic Anemometer (GILL Instruments, UK). The gas analyzer and anemometer were mounted at nine m above ground, approximately four m above the canopy.

Temperature, relative humidity, precipitation, wind and global radiation (350–1500 nm, Rg) were measured using standard equipment approximately 300 m from the flux measurements, using a separate climate station. Additional measurements at this station included net radiation (NR-Lite, Kipp and Zonen), incoming photosynthetic photon flux density (PPFD, 400–700 nm, JYP 1000, SDEC, France), soil moisture at seven levels (TDR, CS615/CS616, Campbell Scientific), soil temperature (soil temperature probe 107/108, Campbell Scientific) at three levels, and soil heat flux (HFP01 Heat Flux Plate, Hukseflux, Delft, The Netherlands). All data were stored at 30 min. averages using a CRX5000 logger (Campbell Scientific). The dry season data covers 12 days (JD 35–46, February 2005) and the wet season data covers eight days (JD 266–273, September 2005), but with 27 missing observations due to computer failure, probably caused by overheating.

### Flux data processing

Fluxes of CO_2 _($F_{CO_{2}}$, *μ*mol *CO*_2 _*m*^-2 ^*s*^-1^), H_2_O ($F_{H_{2}O}$, *mmol H*_2_*O m*^-2 ^*s*^-1^), latent heat (LE, Wm^-2^) and sensible heat (H, Wm^-2^) were measured with the eddy covariance technique according to the EUROFLUX methodology [70,71]. Measurements were made at 20 Hz and stored as 30-min averages. Fluxes from the atmosphere to the biosphere are denoted as negative. The missing data for the wet season (27 samples out of 48*8 samples) were gap filled using the mean values for each corresponding 30 min. period. There were no missing data for the dry season. Spikes > ± 2 standard deviations were removed and replaced with the mean of previous and next 30 min value. This only occurred for two samples, both in the dry season. The data illustrating the dry to wet season transition in 2007 (Figure 8) originate from the same instruments at the same site as the other flux data and were processed in the same way.

### Night time fluxes

In order to cope with reduced $F_{CO_{2}}$ efflux rates at stable atmospheric conditions during night, the relationship between the friction velocity (u*) and the night time $F_{CO_{2}}$ was examined. A clear increase of $F_{CO_{2}}$ with u* was found for the wet season and a weaker increase for the dry season. Hence, night time CO_2 _fluxes at friction velocities ≤ 0.2 *m s*^-1 ^were replaced by the mean night time flux at friction velocities > 0.2 *m s*^-1^. This flux was 1.35 (sd = 0.9) *μ*mol CO_2 _m^-2 ^s^-1 ^for the wet season and 0.27 (sd = 0.14) *μ*mol CO_2 _m^-2 ^s^-1 ^for the dry season.

### Gross primary production, ecosystem respiration and light response curves

Net ecosystem exchange (NEE, *μ*mol CO_2 _m^-2) ^was partioned in gross primary production (Pg, *μ*mol CO_2 _m^-2^), ecosystem respiration (Re, *μ*mol CO_2 _m^-2^), and storage change (Sc, *μ*mol CO_2 _m^-2^) in the air column between the LI-7500 and the ground. Daytime Pg was estimated as F_*C *_– Re and night time Pg = 0. Daytime Re was estimated using soil temperature according to Lloyd and Taylor [72] and night time Re equals $F_{CO_{2}}$. NEE was calculated as [73]:

(1) $$\text{NEE}={\text{F}}_{\text{C}}+{\text{S}}_{\text{C}}={\text{F}}_{\text{C}}+{\text{z}}_{\text{m}}\bar{\rho \text{a}}\frac{\text{d}\bar{\text{C}}}{\text{dt}}$$

where *z*_*m *_is the height above ground for the LI-7500 sensor, $\bar{\rho a}$ is the mean molar density of dry air [mol dry air m^-3^] and $\bar{\text{C}}$ is the mean molar mixing ratio [*μ*mol CO_2 _mol dry air^-1^], $\frac{\text{d}\bar{\text{C}}}{\text{dt}}$ is the temporal difference between $\bar{\text{C}}$ for t and t_-1_.

Light use response curves was created from Pg and PPFD. Ecological LUE, P_*g*_/PPFD, expressed as mmol CO_2 _mol PPFD^-1^, was calculated according to Gilmanov *et al*. [61]. WUE [mmol CO_2 _mol H_2_O^-1^] was calculated as $F_{CO_{2}}$/$F_{H_{2}O}$ and averaged for the dry and wet season data respectively.

### Energy balance

Energy budget closure is a good and independent quality check of eddy covariance measurements due to the similarity of the atmospheric transport mechanism and the theoretical assumptions for all scalars measured with the eddy covariance system (CO_2_, water and heat) [50]. The available energy (net radiation – soil heat flux, Rn-G) was therefore compared to the dissipated energy (H+LE). The soil heat flux (G) was calculated as the mean (of two plates) measured soil heat flux at 7 cm depth minus the storage in the upper 7 cm. We assumed a volumetric heat capacity of 1.28·10^6 ^Jm^-3 ^K^-1 ^and used the measured soil temperature at 3 cm (mean of two sensors) to derive the temperature difference at 3 cm.

## Competing interests

The authors declare that they have no competing interests.

## Authors' contributions

JA conducted the main part of the analysis and wrote the manuscript. MM calculated the energy budget and significantly contributed to data collection, data processing and to the text. BA and HA contributed substantially to data collection, site description and also contributed to the text.

## Supplementary Material

Additional file 1 wet Season. The flux site Demokeya, Sudan during the wet season 2005. Click here for file

Additional file 2 Dry Season. The flux site Demokeya, Sudan during the dry season 2005. Click here for file
