# Supplementary figures and images for: Seasonal variation of carbon fluxes in a sparse savanna in semi arid Sudan
Source: Carbon Balance Manag. 2008 Dec 1;3:7. doi: 10.1186/1750-0680-3-7 (PMC2632635; doi:10.1186/1750-0680-3-7)

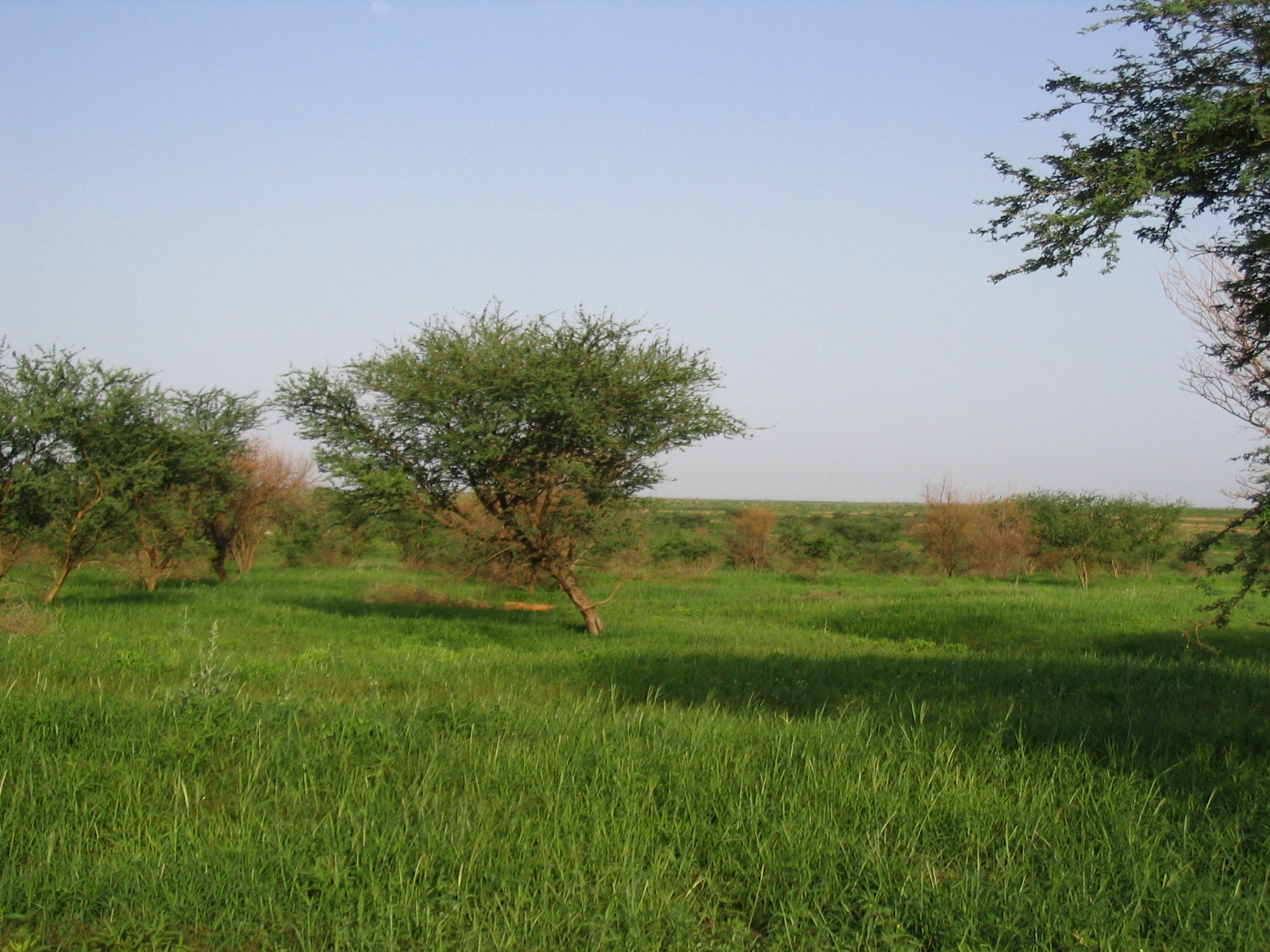

Supplement: Additional file 1 — wet Season. The flux site Demokeya, Sudan during the wet season 2005. [file 1750-0680-3-7-S1.jpeg]

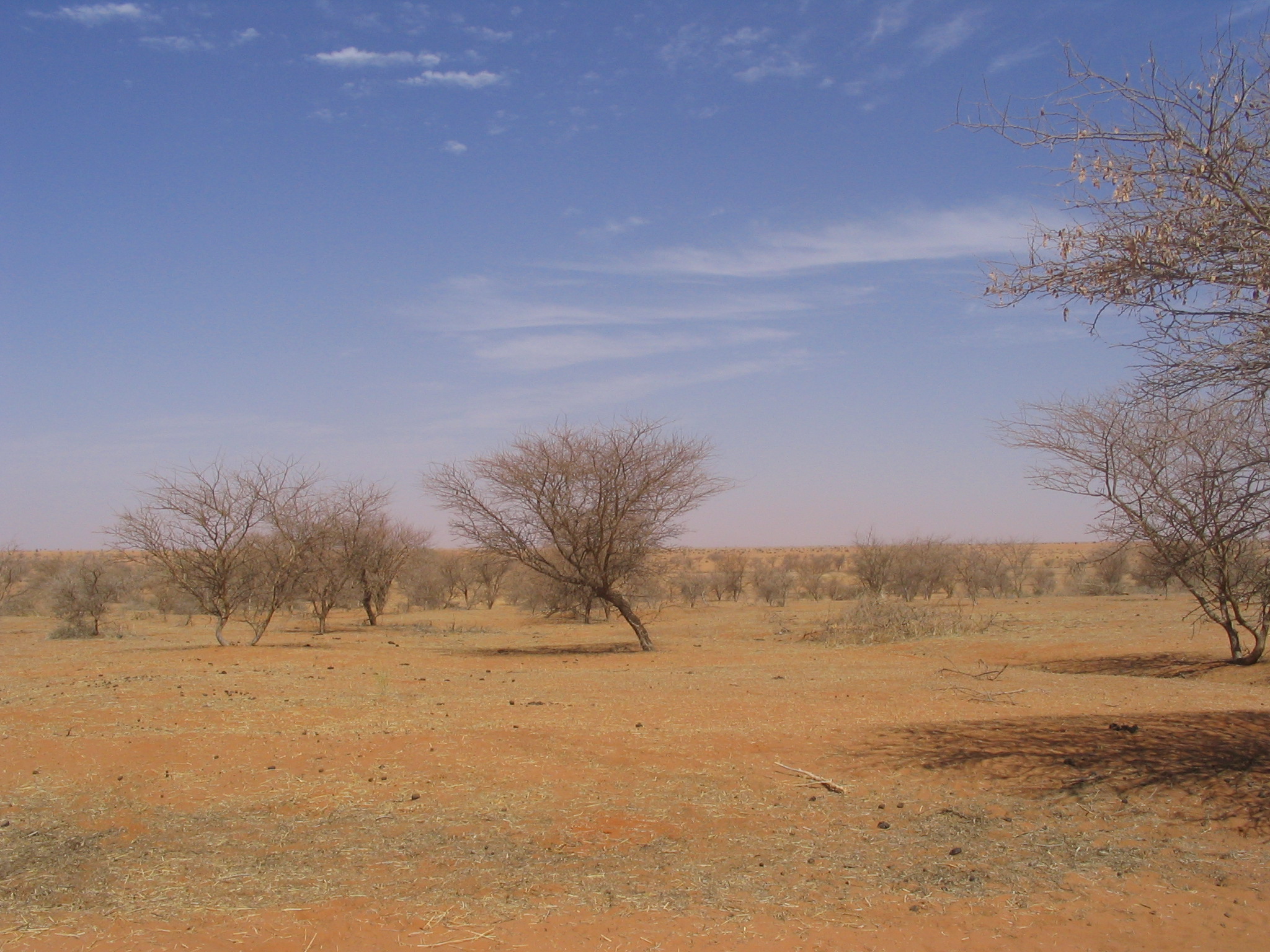

Supplement: Additional file 2 — Dry Season. The flux site Demokeya, Sudan during the dry season 2005. [file 1750-0680-3-7-S2.jpeg]
